# Evaluation of Corneal and Lenticular Parameters in Iron Deficiency Anemia

**DOI:** 10.14744/bej.2021.65477

**Published:** 2021-09-27

**Authors:** Hasan Kiziltoprak, Ali Mert Kocer, Turgay Fen, Yasin Sakir Goker

**Affiliations:** 1. Department of Ophthalmology, Bingol Women’s Health and Children’s Hospital, Bingol, Turkey; 2. Department of Ophthalmology, Health Science University Ulucanlar Eye Training and Research Hospital, Ankara, Turkey; 3. Department of Internal Medicine, Health Science University Ankara Training and Research Hospital, Ankara, Turkey

**Keywords:** Cataract, corneal densitometry, iron deficiency, lens densitometry

## Abstract

**Objectives:**

The aim of this study was to investigate the effects of iron deficiency anemia on corneal and lenticular densitometry.

**Methods:**

Thirty-two patients with iron deficiency anemia and 38 healthy participants were enrolled. The Pentacam HR imaging system (Oculus Optikgeräte GmbH, Wetzlar, Germany) was used to record keratometry, corneal densitometry (12-mm corneal diameter), lens densitometry measurements. Endothelial parameters were evaluated using specular microscopy.

**Results:**

The corneal densitometry values in the anterior 0–2 mm and 2–6 mm zone were significantly higher in the iron deficiency anemia group than in the control group (p=0.044 and p=0.021, respectively). There was a statistically significant difference in the mean values of the standard deviation and maximum lens densitometry measurements of the iron deficiency anemia group when compared with the control group (p=0.012 and p=0.011, respectively). There were statistically significant correlations between the anterior 2-6 mm zone corneal densitometry and ferritin, iron, and total iron binding capacity (r=-0.275, r=-0.243, r=0.240, respectively). However, ferritin, iron, and total iron binding capacity showed no significant correlation with the lens densitometry values (p>0.05 for all).

**Conclusion:**

Iron deficiency anemia had several effects on corneal and lenticular densitometry measurements. Evaluation of the corneal and lenticular changes at an ophthalmology clinic might be recommended for patients with iron deficiency anemia.

## Introduction

Anemia which is defined as blood hemoglobin (Hb) or hematocrit concentration below the lower limit of the normal range is a major and widespread public health problem (1). Iron deficiency anemia (IDA) is the most common cause of anemia among children, women of childbearing age, and pregnant women worldwide (1-3).

The Hb that comprises iron plays an essential role in transporting oxygen from the lungs to the other tissues. Iron also plays important additional roles in the central nervous system, including normal myelination, neurotransmitter synthesis, and neurometabolism (4). In iron deficiency, hypoxia is inevitable as the blood has no longer enough capacity to transport the oxygen to the entire body. As a consequence of hypoxia, detrimental effects can be seen in various systems of the body including ocular structures through enhancement of effects of ischemia (5). Several studies have shown the effect of iron deficiency on ocular structures, especially focused on the posterior segment of eye (6-10). However, a recent study evaluating the anterior segment focusing on endothelial cells showed significant changes at IDA (9). Another study evaluating the risk factors for presenile cataract also found a positive correlation with iron deficiency (10). However, these studies were limited and did not compromise entire anterior segment structures.

There have been important developments in Scheimpflug topography systems that provide quantitative measurements of the clarity of the cornea and lens. The Pentacam HR (Oculus, Wetzlar, Germany) can image the entire anterior segment starting from anterior corneal surface to the posterior lens surface by means of a rotating Scheimpflug camera. That produces anterior and posterior corneal topographic maps and enables three-dimensional analyses of the anterior chamber (11). Another advantage of that imaging system is as it provides quantitative, objective and reproducible corneal and lenticular densitometric data about corneal clarity and lens clarity (12, 13). The Pentacam HR’s densitometry analysis software also measures the intensity of backscattered light from different regions of the cornea and lens (13).

In the present study, we aimed to investigate whether iron deficiency affects the anterior segment parameters of the eye by comparing the results with those obtained in healthy population.

## Methods

This prospective cross-sectional study was performed in the Department of Ophthalmology and Internal Medicine Clinic of two tertiary hospital, between April and October 2019. The study protocol was approved by the Ethics Committee and the study was adhered to the tenets of the Declaration of Helsinki. Informed written consent was obtained from the participants before their admission into the study.

Patients with IDA who were consecutively referred to our ophthalmology clinic from the Internal Medicine Clinic were enrolled as study group. The diagnosis and definition of IDA included all of the following laboratory criteria; Hb <10.5 g/dL; mean corpuscular volume <70 fL; mean corpuscular Hb <23 pg; and mean corpuscular Hb concentration <30 g/dL) and their iron status (serum ferritin level <12 ng/mL and serum transferrin saturation <10%). Age- and sex-matched control subjects (control group) who were randomly selected after a general screening at the ophthalmology outpatient clinic and who had Hb levels of >12 g/dL, ferritin levels of >12 ng/mL, and transferrin saturation >10% were also included in the study. All the control subjects were healthy, without any systemic or ocular disease. Only right eye of each subject was analyzed in the study.

The inclusion criteria were no previous known corneal and lenticular changes; no ocular problems other than spherical or cylindrical refractive errors ≤1.00 diopter (D); best-corrected visual acuity (BCVA) ≥20/20 according to Snellen chart; and no systemic disease, except for newly diagnosed IDA. Participants with the following criteria were excluded; history of glaucoma, keratoconus, corneal dystrophy, lenticular or other ocular media opacities, cataract, contact lens use, ocular trauma, ocular surgery, any chronic disease or uveitis, amblyopia, strabismus detected on ophthalmologic examination; myopic and hypermetropic and astigmatic refractive errors of >1.0 D; drug consumption, smoking, and alcohol consumption. Finally, patients who were uncooperative for Scheimpflug system examinations were also excluded from the study.

All subjects underwent a thorough ophthalmologic evaluation, including assessment of refractive error, BCVA using the Snellen chart (20 feet), intraocular pressure measurement with a pneumotonometer, slit lamp examination and dilated fundoscopy with a 90 D lens. Refraction measurements were performed using the same automatic refractor-keratometer device (Canon RF-K2, Japan).

The corneal examination was performed using a specular microscope (Topcon SP–1P, Japan) and Pentacam HR Scheimpflug imaging system. All the measurements by specular microscope and Pentacam HR Scheimpflug imaging system were performed by the same experienced (masked) clinician under standard dim-light conditions and at the same time of day (between 10.00 and 12.00 am). All the measurements were repeated 3 times per eye, and the one with the best alignment and fixation was chosen for the statistical analysis. Distorted images caused by high reflection and low-quality images that are difficult to evaluate were not included in the analysis.

To evaluate endothelium, subjects were asked to look at the central fixation target and the auto-alignment function of the specular microscopy that was used. Endothelial cell density, average cell area, coefficient variation of cell area (CV) which is standard deviation divided by the mean cell area, and percentage of hexagonal cells (HEX) were calculated by the software of the specular microscope. The co-efficient variation of cell area in cell size was used as an index of polymegethism (the extent of variation in the cell area), and the percentage of HEX in the analyzed area was used as pleomorphism (an index of variation in cell shape).

Pentacam HR measurements were performed first without pupil dilatation to evaluate the keratometry and corneal densitometry values, and second after a pupil dilatation to evaluate the lens densitometry and thickness values, consecutively. Kmax (steepest keratometry on the anterior surface of the cornea), thinnest corneal thickness, flat K (K1) and vertical K (K2) for central 3.0 mm, anterior chamber depth (ACD) was evaluated as anterior segment parameters.

Corneal densitometry measurements (12-mm corneal diameter) were done using densitometry software of the Pentacam HR system. This analysis yielded densitometric values of the cornea at three different depths consisting anterior (120-μm thick - the superficial region of the cornea), central (located between anterior and posterior layer), and posterior layer (60-μm thick - the innermost region of the cornea). This corneal area that was measured was further divided into four concentric zones: The annular area comprised in diameter of 0–2 mm, 2–6 mm, 6–10 mm and 10–12 mm evaluated as first, second, third, and fourth zones, respectively. The corneal densitometry values are expressed as the pixel luminance per unit volume in the scheimpflug image and they were expressed in gray scale units. According to the degree of backscattering light from the cornea, the measurements ranged from 0 (maximum transparency) to 100 (completely opaque cornea).

Lens densitometry values were evaluated after Pentacam HR measurements with dilated pupils which were administered by three drops of 1% cyclopentolate hydrochloride. The measurements were taken approximately 45 min after the last drop. Three-dimensional scan modes were used to measure the density of the lens. The densitometry values in the densitometry zones of the Pentacam HR system were analyzed. The mean value was calculated in predefined 3-D zones (zone 1: 2.0 mm, zone 2: 4.0 mm, and zone 3: 6.0 mm), which were located around the center of the pupil.

### Statistical Analysis

The data obtained from the study were analyzed using the Statistical Package for the Social Sciences (SPSS), version 22.0 for Windows (SPSS Inc., Chicago, IL). Descriptive statistics were presented as the mean ± standard deviations, frequency distributions, and percentages. The normal distribution of the variables was tested using visual (histogram and probability graphs) and analytical methods (Kolmogorov–Smirnov/Shapiro–Wilk Test). Independent samples t-test was used for data with normal distribution, and Mann–Whitney U-test was used for those without normal distribution. Correlation analysis was performed with Spearman’s correlation test. All differences associated with a chance probability of 0.05 or less were considered statistically significant.

## Results

The study included 70 eyes of 70 subjects: 32 of the participants were in the IDA group (29 females and three males), and the remaining 38 (32 females and six males) were in the control group. The mean age of the patients with IDA and that of the matched controls was 41.4±8.7 years and 43.7±10.2 years, respectively. There was no significant difference in terms of age and gender distribution among the groups (p>0.05). The demographic, laboratory results of both groups are shown in Table 1.

**Table 1.** Demographic, laboratory, and ocular characteristics of the groups

	**IDA Group**	**Control Group**	p*
	**(n=32)**	**(n=38)**	
Males/females	3/29	6/32	0.460^¥^
Age, years	41.4±8.7	43.7±10.2	0.704*
BCVA, Logmar	0.1±0.05	0.1±0.04	0.840*
Hemoglobin, g/dL	10.0 ±1.6	13.4±1.3	<0.001*
MCV, fL	75.2±14.7	83.6±6.6	0.002*
Iron, mcg/dL	25.5±7.0	81.7±10.1	<0.001*
Ferritin, ng/mL	8.4±1.5	24.0±6.8	<0.001*
TIBC, mcg/dL	514.3±41.9	306.5±35.6	<0.001*

BCVA: Best corrected visual acuity; TIBC: Total iron binding capacity; MCV: Mean corpuscular volume. ^¥^Chi-squared test. ^*^Independent samples t-test. Bold values indicate p<0.05.

The corneal keratometry values and specular microscopic measurements are shown in Table 2. There was not any statistically significant difference between groups regarding keratometry values and specular microscopic measurements.

**Table 2.** Comparison of the keratometry, anterior segment parameters and specular microscopic measurements in both groups

	**IDA Group**	**Control Group**	p*
	**(n=32)**	**(n=38)**	
K_1_, (D)	42.6±1.5	43.2±1.4	0.140
K_2_, (D)	43.7±1.6	44.1±1.4	0.294
K_max_, (D)	44.2±1.8	44.6±1.5	0.260
Thinnest, μm	535.0±36.6	533.6±42.3	0.879
ACD	2.91±0.3	2.94±0.3	0.660
CCT (μm)	521.2±31.0	534.6 ±43.6	0.213
ECD (cell/mm^2^)	2529.4±186.24	2553.0±220.0	0.676
AVG	397.5±31.1	394.9±35.2	0.777
SD	161.6±39.5	165.7±34.2	0.678
CV	40.3±7.2	41.5±6.5	0.511
HEX	44.2±4.9	42.6±6.1	0.312

BCVA: Best corrected visual acuity; ACD: Anterior chamber depth; CCT: Central corneal thickness; ECD: Endothelial cell density; AVG: Average cell area; SD: Standard deviation CV: Co-efficient of variation of cell area; HEX: Percentage of hexagonal cells. *Independent samples t-test. Bold values indicate p<0.05.

The corneal densitometry values of the groups measured by Pentacam HR are shown in Table 3. The densitometry at anterior 0–2 mm and 2–6 mm zone was significantly higher in IDA group than control group (p=0.044 and p=0.021, respectively).

**Table 3.** Comparison of the corneal densitometry measurements (gray scale units) in both groups

	**IDA Group**	**Control Group**	p*
	**(n=32)**	**(n=38)**	
Anterior 120 μm			
0-2 mm	16.6±1.3	16.0±1.0	**0.044**
2-6 mm	15.3±1.4	14.6±1.0	**0.021**
6-10 mm	17.6±4.4	16.2±2.8	0.120
10-12 mm	26.5±8.1	26.8±8.1	0.856
Total Diameter	18.1±2.9	17.4±2.0	0.216
Center			
0-2 mm	11.2±0.9	11.1±0.8	0.483
2-6 mm	10.4±1.1	10.1±0.8	0.244
6-10 mm	12.5±3.1	11.6±2.0	0.188
10-12 mm	16.8±4.5	16.7±3.7	0.883
Total Diameter	12.3±2.0	11.8±1.3	0.291
Posterior 60 μm			
0-2 mm	8.7±1.1	8.5±0.9	0.340
2-6 mm	8.3±1.2	8.0±0.9	0.287
6-10 mm	10.6±2.6	9.8±1.5	0.142
10-12 mm	13.5±3.3	13.0±2.2	0.487
Total Diameter	10.0±1.8	9.5±1.1	0.189
Total thickness			
0-2 mm	12.2±1.0	11.9±0.9	0.148
2-6 mm	11.3±1.2	10.9±0.8	0.106
6-10 mm	13.5±3.3	12.6±2.0	0.139
10-12 mm	18.9±5.0	18.8±4.3	0.940
Total Diameter	13.5±2.2	12.9±1.4	0.224

*Independent samples t-test. Bold values indicate p<0.05.

The lens densitometry values in the IDA and control groups are shown in Table 4. Mean values of the standard deviation and maximum lens densitometry measurements of the IDA group were significantly higher than control groups (p=0.012 and p=0.011, respectively). Although the lens densitometry values of zone 1, zone 2, and zone 3 were higher in the IDA group, these differences were not statistically significant between the groups (p>0.05, for all).

**Table 4.** Comparison of the lens densitometry measurements and lens thicknesses in both groups

	**IDA Group**	**Control Group**	p*
	**(n=32)**	**(n=38)**	
Zone 1	8.5±0.9	8.4±0.6	0.764
Zone 2	8.0±0.4	7.9±0.4	0.809
Zone 3	7.9±0.4	7.7±0.2	0.190
Average	8.7±1.1	8.7±0.8	0.994
Standard deviation	3.9±2.5	2.5±1.8	0.012
Maximum LD	49.0±28.5	34.1±16.1	0.011

LD: Lens densitometry. *Independent samples t-test. Bold values indicate p<0.05.

**Figure 1.**
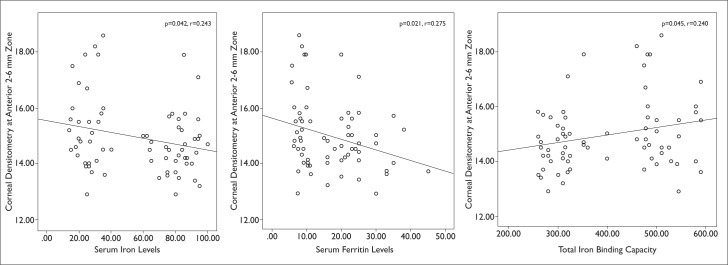
Correlation of ferritin, iron and total iron binding capacity (TIBC) with anterior 2-6 mm zone corneal densitometry showed significant correlations.

When we correlate ferritin, iron and total iron binding capacity (TIBC) with lens densitometry measurements, there were statistically significant correlations between the anterior 2–6 mm zone corneal densitometry and all these three values as illustrated in Figure 1 (r=–0.275, r=–0.243, r=0.240, respectively). However, there was not any statistically significant difference with the other zones (p>0.05, for all). Ferritin, iron and TIBC showed no significant correlation with lens densitometry values (p>0.05, for all). Moreover, none of the corneal keratometry values and specular microscopic values were significantly correlated with ferritin, iron and TIBC levels (p>0.05, for all).

## Discussion

In the present study, we investigated whether corneal clarity and lens clarity affected in patients with IDA by measuring corneal and lens densitometry values. We demonstrate that patients with IDA had a significantly higher corneal densitometry values at anterior 0–2 mm and 2–6 mm zone, mean values of the standard deviation and maximum lens densitometry measurements.

IDA is the most frequent and treatable anemia worldwide (1-14). The prevalence of IDA is higher in women, especially in premenopausal women and uncommon in adults (1, 2, 14). In Turkish population, nearly half of the women in the childbearing age had anemia and IDA was the most frequent one (15, 16). In our study, the participants were mostly women, which is not surprising, as IDA is more common among them.

Iron is an essential component of Hb in order to transport oxygen to the peripheric tissues. In IDA, Hb has no longer enough capacity for transporting the oxygen and hypoxia is the rule (5). The hypoxia that stems from IDA will lead to changes in many tissues especially those without circulatory system. There is no direct vascular system that feeds the cornea and lens, and they are fed by diffusion from the aqueous and vitreous. Therefore, these tissues are more prone to ischemic changes than other tissues.

When we evaluate anterior segment for keratometric and anterior chamber variables, there was not any significant change between groups. The hypoxia resulted in IDA may affect corneal mechanics and ACD by the ischemia of the cells that produces aqueous humor. However, we observed no difference between groups regarding these values. On the other hand, when we evaluate corneal clarity by corneal densitometry measurements, significant changes were observed, at anterior 0–2 mm and 2–6 mm zone of the cornea. The other zones and layers were same as the controls. Moreover, it was found that ferritin and iron levels were inversely correlated with the densitometric values of anterior 2–6 mm zone of the cornea. In addition, TIBC, whose greater levels are related to the IDA, was positively correlated with the densitometric values of anterior 2–6 mm zone of the cornea. Considering that the cornea is fed by aqueous, the change in the anterior layers may be attributed that the anterior layer is far from the aqueous when compared with the other layers and more prone to ischemia. The densitometric values at the other zones of anterior layer were similar as controls. It is well known that the epithelium, which is the first 50 microns of anterior cornea, receive oxygen from conjunctival capillaries through tear film. The significant alteration in central zones of anterior layer could also be explained by their distance from the conjunctival capillaries. Corneal clarity is crucial for the precise function of the cornea. The ischemia caused by IDA affects the corneal clarity at anterior zone, which may be a beginning for the opacification of the cornea.

Lens is another avascular component of the eye that needs high level of oxygen for precise visual function. A study evaluated risk factors for presenile cataract, iron deficiency status was found as a compounding factor for the early opacification of the lens (10). In that study, higher TIBC level which is related to iron deficiency was associated with cataract (10, 17, 18). In addition, they found that the iron supplementation may reduce oxidative stress (19). In our study, when we evaluate the lens densitometry measurements by Pentacam HR, mean values of the standard deviation and maximum lens densitometry measurements were found significantly different from the controls. However, the lens densitometric values of three zones were higher in the IDA group; these differences were not significant between the groups. These results suggest that lens clarity decreased in the patients with IDA. The ischemia that induced by IDA caused deterioration in the structure of the lens fibers and cataract could develop, resultantly. By the deepening of the ischemia the oxidative stress will also be a contributing factor for that formation. The iron supplementation will decrease the ischemia and oxidative stress and may reverse the formation of the cataract when given early periods. On the other hand, a recent study found a significant relation between higher TIBC and presenile cataract (10). While we evaluated the correlation of ferritin, iron and TIBC with lens densitometry measurements, we found no significant difference.

Corneal transparency plays a critical role in vision and it is provided by extensive metabolic activity of endothelial cells. Endothelial cells need high amount of oxygen to continue that metabolic activity. Coskun et al. (9) found that endothelial functions are affected in patients with IDA. They also found higher CV and lower HEX in IDA group than healthy individuals in another study (20). In our study, we found no significant changes in endothelial values and central corneal thickness. The hypoxic environment in the IDA subjects may involve corneal endothelium. However, the amount of ischemic process may be important for that involvement. The normal endothelial function in our study may be the result of the same reason that the only anterior layers of the corneal densitometry affected by the ischemia in the anterior chamber.

The current study had several limitations. First of all, our study group consisted of a relatively small number of patients. Second, we did not know the duration of IDA prior to the study. However, it is not possible to know the duration of anemia precisely. That is why we can’t speculate how long it takes to change the corneal and lenticular changes. Finally, our study has a cross-sectional nature, the causal relationship between IDA with corneal and lenticular changes cannot be inferred. Further studies with high number of patients may detect significant changes in the corneal and lenticular densitometry values. Since corneal hypoxia due to contact lens use may develop, excluding patients with contact lens use in this study enabled us to better evaluate the results of our study, and this was one of the strengths of our study.

## Conclusion

IDA has several effects on the corneal and lenticular densitometry measurements. However, the effects of IDA on the endothelium and keratometric values were not significant. Patients with IDA might be consulted to ophthalmology clinic for the evaluation of the corneal and lenticular changes.

## Disclosures

### Ethics Committee Approval:

Ankara Training and Research Hospital Clinical Research Ethics Committee, protocol number: E-19, Date: 05/12/2019.

### Peer-review:

Externally peer-reviewed.

### Conflict of Interest:

None declared.

### Authorship Contributions:

Involved in design and conduct of the study (HK, AMK, YSG); preparation and review of the study (HK, YSG, TF); data collection (HK, TF, AMK); and statistical analysis (HK, AMK, YSG).
